# CoVnita, an end-to-end privacy-preserving framework for SARS-CoV-2 classification

**DOI:** 10.1038/s41598-023-34535-8

**Published:** 2023-05-08

**Authors:** Jun Jie Sim, Weizhuang Zhou, Fook Mun Chan, Meenatchi Sundaram Muthu Selva Annamalai, Xiaoxia Deng, Benjamin Hong Meng Tan, Khin Mi Mi Aung

**Affiliations:** Institute for Infocomm Research, Agency for Science, Technology And Research (A*STAR), Singapore, Singapore

**Keywords:** Computational science, Classification and taxonomy, Machine learning

## Abstract

Classification of viral strains is essential in monitoring and managing the COVID-19 pandemic, but patient privacy and data security concerns often limit the extent of the open sharing of full viral genome sequencing data. We propose a framework called CoVnita, that supports private training of a classification model and secure inference with the same model. Using genomic sequences from eight common SARS-CoV-2 strains, we simulated scenarios where the data was distributed across multiple data providers. Our framework produces a private federated model, over 8 parties, with a classification AUROC of 0.99, given a privacy budget of $\varepsilon =1$. The roundtrip time, from encryption to decryption, took a total of 0.298 s, with an amortized time of 74.5 ms per sample.

## Introduction

The pathogenic virus known as SARS-CoV-2 emerged quietly in the final months of the year 2019 in Wuhan, in the Hubei province of China. The virus caused severe respiratory symptoms in patients and was highly transmissible, spreading across the globe within weeks. By 31st January 2020, the World Health Organization had declared a public health emergency on COVID-19, the disease caused by SARS-CoV-2. As the third pandemic of the 21st century, COVID-19 placed significant stress not just on the global healthcare infrastructure, but also on the global supply chain networks. Although significant discoveries in COVID-19 vaccines and anti-viral treatment options have helped restore a semblance of normality in several developed countries, COVID-19 remains a threat globally two years into the pandemic. Part of the difficulty in stemming the tide, or eradicating the virus, is due to the high mutation rate of the virus. The pandemic has been marked by waves of new infections and reinfections caused by the rise of newer strains of the virus: first the alpha and beta strains at the end of 2020, which were then displaced by the more transmissible delta strain, and eventually the current dominant strain, omicron. Given the speed at which novel coronavirus variants develop in different geographical pockets around the world, there is a pressing need for the development of infrastructure to perform global surveillance and outbreak prediction. To this end, initiatives such as the Global Initiative on Sharing Avian Influenza Data (GISAID) have played an important role in monitoring the pandemic.

Yet, privacy concerns have been raised regarding the sharing of viral sequencing data, as such data can be used in tandem with other contextual clues to establish patient identity. As the virus spreads mainly by close contact, the circulation of a new viral strain within groups of individuals may indicate that they have engaged in social activities together. This can lead to ostracization or discrimination, especially if there is a social stigma associated with the disease within the community. For instance, one of the COVID-19 clusters in South Korea had been linked to members of a religious cult^1^, while one of the largest COVID-19 waves in Singapore was linked to socializing between karaoke patrons and sex workers^2^. In both cases, the patients were accused of not following public health guidance in place at that time, and for acting irresponsibly and selfishly. Although they were not the only patients who had contracted COVID-19 during that period, they could be identified because of the strains they were carrying. To assuage the privacy and security concerns of institutions and individuals regarding such crucial information, care must be taken to ensure that the confidentiality of the data is not compromised during any downstream analysis which is achieved with our proposed framework, CoVnita, by obfuscating samples and the classification outcome with HE, preventing unintended negative consequences.

The Integrating Data for Analysis, Anonymization and SHaring (iDASH) centre organizes the annual Secure Genome Analysis Competition to bring together bioinformaticians and cybersecurity experts to address problem statements regarding privacy concerns arising from data sharing in bioinformatics research. In 2021, Track II of the competition posed the pertinent challenge statement: to detect and track viral strains, SARS-CoV-2 viral samples have to be classified as one of many known strains. However, the sample data cannot be shared due to policies put in place to protect patient privacy.

In this paper, we present a framework to first enable the private training of a model and then secure classification using the said model. Note that the secure classification technique described here was the winning solution submitted by our team (A*FHE-2) to Track II of the iDASH 2021 competition^3,4^. Beyond demonstrating the analysis of genomic data privately and securely, we have also performed additional analysis to assess the eroding effect of differential privacy and data variability on model performance. Our proposed framework achieves the following:

- Enables different organizations to jointly train a model securely end to end, preserving the privacy and confidentiality of patients’ data from training to inference.
- Provides quick and accurate classification of COVID-19 strains to improve triage of patients based on the predicted outcomes of the classified strains, thereby alleviating the burden on hospital infrastructure.

## Results

**Figure 1:**
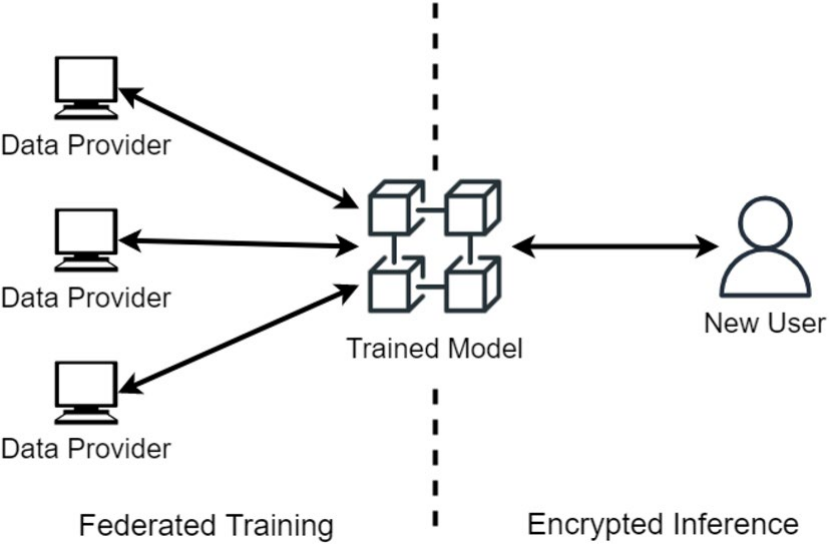
Outline of CoVnita. The training phase begins with the data providers each locally training a differentially private local model. A federated feature selection is first performed to reduce the size of the data while maintaining its quality. Next, they share and compute a global average of their local models. This process of local updates and joint averaging then repeats for a fixed number of epochs. Throughout this process, a joint global model, that does not require data providers to share their genomic samples, is trained. To ensure the robustness of the model against membership inference attacks, differentially private noise is injected during the training process. The classification process is performed in its entirety with the samples encrypted with HE, a special type of encryption that supports computation on encrypted data. This means that new samples can be evaluated in their encrypted form, ensuring that the data stays private. An efficient HE packing method was used, allowing many samples to be simultaneously classified.

CoVnita, Fig. 1, provides an end-to-end workflow that trains a model across multiple parties securely with Federated Learning (FL), further reinforced by injecting Differential Privacy (DP), and classifies new samples privately with Homomorphic Encryption (HE). An honest-but-curious threat model is assumed in this work. This means that the parties in the protocol will adhere to the protocol, but are curious about another party’s private information. All communication channels between parties are assumed to be secure.

### Privacy-preserving model training

We demonstrate our framework on eight COVID-19 variants that had gained prominence at various time points during the pandemic, including four that were provided in the iDASH 2021 competition^4^. We characterize the quality of the genomic data from these sequences in Table 1.

**Table 1:** Distribution of nucleotide bases.

PANGO lineage	Variant	A	T	C	G	N	Others
*B*.1.617.2	Delta	17,350,073	18,673,551	10,640,782	11,396,199	1,435,543	1618
*C*.37	Lambda	17,678,438	19,008,782	10,830,235	11,585,368	404,444	1522
*B*.1.621	Mu	17,324,855	18,658,329	10,624,188	11,385,289	1,439,488	1168
*B*.1.1.529	Omicron	17,359,295	18,659,613	10,643,562	11,405,345	1,426,693	551
*B*.1.429	Epsilon	17,658,000	18,978,000	10,838,000	11,596,000	494,000	0
*B*.1.1.7	Alpha	17,698,533	19,045,276	10,868,253	11,617,247	404,750	542
*P*.1	Gamma	17,740,786	19,086,620	10,892,220	11,648,508	300,750	417
*B*.1.526	Iota	17,699,086	19,043,095	10,867,848	11,618,684	421,301	326

The percentage of uncertain bases (*N* and *Others*) in the sequences ranges from 0.5 to 2.5%, with the Gamma and Mu variants having the highest and lowest quality sequences respectively.

The sequences were reduced to a more compact set of hashed features using a technique called Dashing (see “Methods”). For more efficient training and inference, we perform a round of federated feature selection using Fed-$\chi ^2$^5^ to approximate the top 15 informative features. Then, we apply FL to enable data contributors to jointly build the virus strain classification model without revealing their data. DP was applied to further enhance the privacy of the dataset by releasing differentially-private local models

Two setups, a balanced and imbalanced data split, were used to evaluate the model training framework. The first setup involved balanced combinations where the data is split evenly over 8 parties in various degrees, ranging from 1 up to 8 variants per party. There are multiple permutations for each scenario where each party hold either 1, 2, 4 or 8 variants, with the same number of samples per party (2000). One possible combination is described in Supplementary Tables S1 to S4. This results in differing local models that aggregate into distinct global models. A total of 301 possible configurations were tested, 100 for each of the scenarios with 1-, 2- or 4-variants and 1 for the 8-variant case. These were generated randomly and the average performance for the different variant scenarios was recorded. The second setup considered two different types of imbalanced data splits that may be more applicable to real-world scenarios. The first imbalanced data split focuses on the 4 variants, *B*.1.617.2 (Delta), *C*.37 (Lambda), *B*.1.621 (Mu), *B*.1.1.529 (Omicron), obtained from the GISAID database, based on their preponderance in different geographical continents, which we described in Supplementary Table S5. In the second imbalance data split, described in Supplementary Table S6, the data was randomly assigned amongst the 8 parties via sampling from a uniform distribution.

To demonstrate the effectiveness of our framework, we also trained a model with the entire dataset to use as the baseline for comparisons with models trained without DP. We present the performance of our models trained with a total of 16,000 samples, 2000 samples per variant. The models were tested with an unseen test set of 4000 samples—500 samples per variant. These results are summarized in Table 2 and their statistical distribution is given in Supplementary Tables S7 to S9. Performance (based on AUROC) between models trained in centralized and federated settings was largely similar.

**Table 2:** Model performance for centralized and federated settings.

Setting	Distribution of samples	# Variants per party	Average accuracy	Average AUROC
Centralized	−	−	0.986	0.992
Federated	Balanced	1	0.873	0.978
		2	0.946	0.995
		4	0.980	0.999
		8	0.984	0.998
	Imbalanced	2–$4^{\star }$	0.944	0.999
		7–$8^{\#}$	0.975	0.998

$(^\star )$ refers to a split configuration based on geographical locations across 6 parties and $(^\#)$ denotes a random split of samples across 8 parties. In the federated setting, there is an increase in average model performance with greater variability of variants that each party holds.

The models were then subsequently enhanced with DP and tested with the same samples. Increasing the amount of noise introduced during the DP-SGD training process leads to a lower privacy budget. Table 3 describes the federated model performance over four different privacy budgets $\varepsilon =0.1,1.0,3,\infty$, in decreasing order of privacy.

The geographical split scenario produced models with the lowest model accuracy (0.338, Table 3) due to the asymmetrical split of the data, and a stronger privacy guarantee in this scenario resulted in larger distortion on an already sparse data split. However, the AUC metric remains relatively high (0.710, Table 3), indicating that the classification of certain variants remains fairly accurate.

Overall, our results suggest that FL with DP is a feasible approach to enable privacy-preserving collaborative machine learning in real-world settings.

**Table 3:** Model performance for federated models with varying $\varepsilon$.

# Variants per Party	$\varepsilon =0.1$		$\varepsilon =1.0$		$\varepsilon =3.0$		$\varepsilon =\infty$ (no DP)	
	Average accuracy	Average AUROC	Average accuracy	Average AUROC	Average accuracy	Average AUROC	Average accuracy	Average AUROC
1	0.716	0.934	0.876	0.985	0.878	0.986	0.873	0.978
2	0.776	0.951	0.943	0.996	0.950	0.996	0.946	0.995
4	0.827	0.966	0.971	0.998	0.972	0.999	0.980	0.999
8	0.691	0.920	0.952	0.994	0.952	0.995	0.984	0.998
2–$4^\star$	0.338	0.710	0.938	0.985	0.982	0.997	0.994	0.999
7–$8^{\dagger }$	0.802	0.958	0.959	0.997	0.963	0.997	0.975	0.998

$(^\star )$ refers to a split configuration based on geographical locations across 6 parties and $(^\#)$ denotes a random split of samples across 8 parties. We observe that model performance generally decreases with the addition of differential privacy and that lowering the privacy budget leads to models with poor performance. Nonetheless, at a reasonable privacy level of $\varepsilon = 1$, there is little to no degradation of the model.

### Homomorphic classification

We used the SEAL library (version 3.2.2)^6^ for HE instantiation with the following parameters: $\log N = 13$, $\log p = 30$, $\log Q = 90$. This gives us a security level of at least 128-bits according to the estimator by^7^. This particular setting allows a ciphertext to support up to 4096 samples. The same test set of 4000 samples that were used to evaluate the federated models was encrypted and used for the Homomorphic Classification.

**Figure 2:**
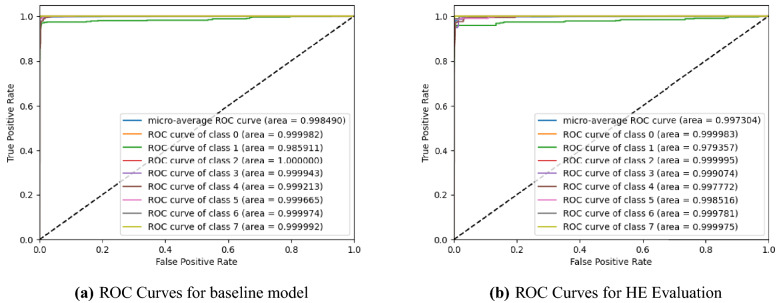
ROC curves for centralized model evaluation with and without HE. There is small to no loss in accuracy when evaluating the model homomorphically.

We report the ROC curves for the baseline model with and without HE in Fig. 2. The various time taken for different processes during the encrypted inference is reported in Table 4 and the amount of storage used during the encrypted classification is reported in Table 5. The short run-time and low storage indicate that there are little to no trade-offs to switching to a HE-based model.

**Table 4:** Time taken for homomorphic inference.

Process	Time taken (s)
Encoding model	0.128
Encrypting 4000 samples	0.044
Homomorphic inference	0.103
Decryption and decoding	0.023
Total time taken	0.298

**Table 5:** Storage consumption of homomorphic evaluation.

Object	Storage per object (kB)	Total storage (MB)
Secret key	385	0.385
Public key	193	0.193
Evaluation key	1200	1.2
Ciphertext	385	5.7
Plaintext	193	25
Total storage		32.5

There is a total of 128 Plaintext objects and 15 Ciphertext objects. See “Methods” for details on deriving the number of Plaintext and Ciphertext objects.

## Discussion

In this work, we have demonstrated how a machine learning model can be trained jointly and securely from several data sources with federated learning and differential privacy, and how inference on new samples can be achieved in a privacy-preserving manner via homomorphic encryption techniques. Data from each owner stays locally on-premise and is never exposed to other owners in the system throughout the whole process from model training to inference. Each data owner will not be able to learn anything about the data from other owners, beyond what can be inferred via the global model. Choosing an appropriate machine learning algorithm for the global model, for instance, logistic regression, will then restrict the sharing of information and prevent the exposure of individual data values. In contrast, machine learning models such as K-nearest neighbour are inappropriate as they will expose all the individual data values. We base our discussion here on a logistic regression model. We note that our model can perform inference tasks directly on encrypted inputs, for instance, on new samples to be classified.

### Comparisons to related work

Several recent developments in the cybersecurity domain have focused on training a model securely with technologies such as homomorphic encryption (HE)^8–10^ and multi-party computation (MPC)^11–13^. Two recent works that presented frameworks using a combination of at least two privacy-preserving technologies have been published by Kaissis et al.^14^ and Carpov et al.^15^.

Specifically, Kaissis et al. introduced a framework called PriMIA (Privacy-preserving Medical Image Analysis), which allows data owners to collaborate and train a medical image classification model securely via FL, utilizing DP which provides an additional layer of privacy. The evaluation of the model is then executed via a 2-party protocol^16^ based on a type of MPC known as Function Secret Sharing, split between 2 servers. This means that, from a security aspect, there is a need to protect the 2 servers from malicious clients. CoVnita, on the other hand, only requires standard cryptographic key management, which is simpler to protect. In addition, the replacing of MPC with HE means that the evaluation phase of our solution does not require a pool of correlated randomness for effective use, and also can be extended to use 2-key HE with techniques described in Chen et al.^17^.

Carpov et al. argue that MPC is a better alternative to FL, as the latter may lead to possible leakage of information about the model during gradient updates. Their proposed framework (GenoPPML) utilizes both MPC and HE, where a logistic regression model is trained with MPC over 2 servers with a differentially private mechanism. New samples to be evaluated are then encrypted with HE before being classified by the model. In our work, we mitigate the information leakage concerns of Carpov et al. regarding federated learning by utilizing differential privacy, thus avoiding the hefty overheads of using MPC for more than 3 parties.

### Enabling privacy-preserving technologies via domain-aware data preprocessing

Despite rapid developments in the cybersecurity space, many of the tools are not optimized to handle “-omics” data, which have far higher dimensionality than data from traditional fields such as image classification. Although GenoPPML^15^ demonstrated feasibility on gene expression data, we note that the largest processed feature space in that work was 25,128. In comparison, the genomic sequence length of the SARS-CoV-2 viral strain here is approximately 30 kB, which would result in more than 90,000 processed features under a standard one-hot encoding schema, assuming the simplest case of limiting encoding to the four nucleotide bases.

It is useful to leverage domain knowledge to make such “-omics”-problems more tractable for privacy-preserving technologies. Good data preprocessing step can help reduce the dimensionality of raw data significantly while retaining important information relevant to the classification task. For instance, our work here has utilized Dashing, a hashing technique commonly used in genome classification, to provide a layer of abstraction from the raw sequence data. Further, we leverage biological knowledge that mutations in the *S* gene (which encodes the spike protein that influences infectivity), are key drivers of biological differences between the strains^18^ to reduce the initial size of the raw data. Specifically, we truncated the first 20 kB of the genome sequence (the regions preceding the S gene) before Dashing and encryption. This allowed us to achieve faster data preprocessing and model training speeds and was used in our submission to the iDASH 2021 competition^4^ that won first place.

### Limitations and future work

While an honest-but-curious threat model is usually sufficient in most situations, we acknowledge that our framework is unable to defend against truly malicious adversaries. A malicious data provider could for instance contribute substandard data that would affect the quality of the trained model. We emphasize that while our current setup does not prevent such acts of vandalism that lead to the degradation of overall model performance, the privacy of data from each owner in the system will not be compromised by this.

Our proposed framework also does not provide proof to an end user that the computation had been performed correctly. For instance, issues arising during deployment may lead to a malfunctioning classifier, but an end user who submits a new sample will be none-the-wiser about this. This is a limitation in HE setups, as visibility on the ’proof-of-computation’ is low or non-existent to the end user.

Although we have simulated a distributed set of data owners in this work, we note that thee also exist centralized resources for SARS-CoV-2, such as GISAID’s EpiCoV platform, that serve as a trusted platform for researchers to share information. Our future work will consider how our technology can add value to such systems that are based on a trusted central platform. Another future work would be to extend this framework to support other models or statistics (e.g. Kaplan–Meier survival analysis) and other forms of medical data (e.g. images).

## Methods

**Figure 3:**
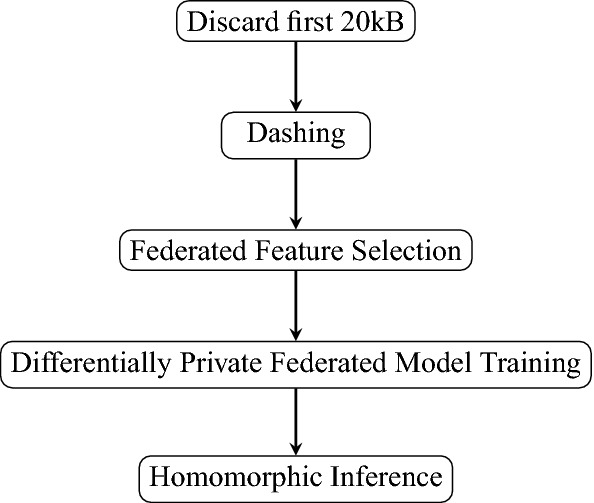
CoVnita workflow. The first 20 kB is first discarded to reduce the size of the data. A tool called Dashing is used to transform the truncated genomic sequence into 512 features, each a 64-bit hash value. The parties perform a federated feature selection to further reduce the number of features to 15. A model is then trained in jointly with FL and DP. The test samples are encrypted and evaluated with HE.

A figure outlining the whole process from data processing to encrypted inference is described in Fig. 3. Here, we provide preliminaries for the key technologies used in our framework and details of each component in the workflow above.

### Federated learning

Federated learning (FL) is a technique in machine learning that allows multiple nodes to train models without exchanging data directly. It was originally developed by Google to train a global model across mobile devices using a central server^19^. Each node possesses a dataset on which they will locally process and provide an update to the global model. More precisely, each node train a local model *w*, over *n* samples, with the following objective function $\min \frac{1}{n} \sum _{i=1}^{n} f_i(w)$, where $f_i(w)$ is usually set to be the loss between the prediction of the *i*-th sample and its actual value. Each node *k* locally computes $g_k = \nabla f_i(w)$. The central server then computes the aggregate of $g_k$ and updates the model, for some fixed learning rate $\eta$, with $w_{t+1} \leftarrow w_t + \eta \sum _k g_k$. In this manner, the raw data is not shared between the nodes or the central server.

Based on the distribution of the data attributes and sample spaces, FL can be categorized into two broad categories—horizontal FL and vertical FL. Horizontal FL refers to each node having similar data attributes, but different sample spaces, while the nodes in vertical FL have different (and often unique) data attributes of the same set of samples. Horizontal FL can be further subdivided into two categories based on the number of data points each node possesses. If all nodes have an identical number of data points, then it is labelled as an independent, identically distributed (IID) distribution and otherwise, a non-IID distribution.

### Homomorphic encryption

Homomorphic encryption (HE) is a special type of encryption scheme that allows computation to be performed on encrypted data. It was first proposed by^20^, with the first construction achieved by^21^. HE schemes are “noisy” in general, where noise is applied to a message as part of the encryption process to mask its value. A noise budget is set when the encryption scheme is initialized and computation (called homomorphic operations) on encrypted data consumes it. Once the noise budget is fully depleted, decrypting the ciphertext would result in an incorrect result.

In this work, we use the CKKS scheme^22^ which supports homomorphic operations on encrypted approximate numbers. Each number is encrypted with an initial precision and computation gradually reduces it. Thus, the decrypted message is an approximation of the true computation result.

Crucial to practical performance, HE schemes can store and simultaneously operate on more data in a single ciphertext by leveraging the decomposition of the plaintext space $R = \mathbb {Z}[x]/\langle x^{N}+1\rangle$^23^. The CKKS scheme supports the encoding of $\frac{N}{2}$ complex numbers into a degree $N-1$ polynomial via the canonical map $\phi : \mathbb {C}^{N/2} \rightarrow R$. This process is described by the following functions:

- $$m(x) = \textsf{Encode}(z_0, z_1, \ldots , z_{N/2-1}) = \phi (z_0, z_1, \ldots , z_{N/2-1})$$.
- $$(z_0, z_1, \ldots , z_{N/2-1}) = \textsf{Decode}(m(x)) = \phi ^{-1}(m(x))$$.

Each number is encoded into a slot of the ciphertext (N/2 many in each). This reduces the number of ciphertexts required in applications and each homomorphic operation is done on all slots in parallel, i.e. adding and multiplying ciphertexts result in the same operation applied to all slots respectively. There is also an inter-slot data movement mechanism that will return a ciphertext whose slots are rotations of those in its input.

### Differentially private stochastic gradient descent

Differential privacy (DP) is a privacy mechanism that protects an individual’s data when it is used in a database. A formal definition proposed by^24^ states that for two datasets *D* and $D'$ differing in at most one record, given an algorithm $\mathcalligra {M}$, we say that $\mathcalligra {M}$ is $(\varepsilon ,\delta )$-differentially private if $\text {IP}[\mathcalligra {M}(D)=x] \le \exp (\varepsilon ) \cdot \text {IP}[\mathcalligra {M}(D')=x] + \delta .$ The parameter $\varepsilon$ can be thought of as the privacy budget or the largest distance between the outputs of $\mathcalligra {M}$ on the datasets. If $\varepsilon =0$, it is equivalent to having different datasets giving the same output. The parameter $\delta$ on the other hand represents the probability of the individual’s data leaking. Differentially private mechanisms have an interesting property of being robust to post-processing. This means that any function applied to the output of any differentially private mechanism is also differentially private.

Stochastic gradient descent (SGD) is an iterative method commonly used in machine learning algorithms. It is used as a low-cost alternative to other second-order methods for finding the local minimum of the objective function, at the expense of a lower convergence rate.

In machine learning, the de facto standard would be to add DP during the model training, specifically to stochastic gradient descent (SGD). This allows the model to be distributed subsequently while ensuring the privacy of the data used for training. Abadi et al.^25^ proposed the following method of applying DP to SGD; First, compute the gradients of the loss function (for each feature). Next, clip the gradients such that the gradient vector has a norm less than some predetermined threshold. Finally, add a suitable amount of Gaussian noise.

### Data and preprocessing

We selected eight COVID-19 strains namely *B*.1.1.7 (Alpha), *B*.1.429 (Epsilon), *P*.1 (Gamma), *B*.1.526 (Iota), *B*.1.617.2 (Delta), *C*.37 (Lambda), *B*.1.621 (Mu) and *B*.1.1.529 (Omicron). For each strain, we obtained 2500 sequences, of which 500 were set aside as a held-out test set for evaluating model performance. The samples for the *B*.1.1.7 (Alpha), *B*.1.429 (Epsilon), *P*.1 (Gamma) and *B*.1.526 (Iota) strains were the same ones provided in the iDASH 2021 competition^4^. The remaining samples for the *B*.1.617.2 (Delta), *C*.37 (Lambda), *B*.1.621 (Mu) and *B*.1.1.529 (Omicron) strains were obtained from the Global Initiative on Sharing Avian Influenza Data (GISAID) database^26–28^ (accessed on 31 Dec 2021).

As viral strains are typically defined based on their phenotypic characteristics rather than simple sequence similarity^29,30^, alignment-free methods^31,32^ are better suited to perform the classification. These methods transform raw genomic sequences into feature vectors that are then used to train machine learning models.

Dashing^33^ is a tool used to estimate the similarities of two genomic sequences. For each genomic sequence, we truncate the first 20 kB and then split the remaining into *k*-mers, where we chose $k=31$, as tested in^33^. Each *k*-mer is then converted into a 64-bit hash. The similarities of the genomic sequences (or equivalently, approximate distance) can then be computed by checking if a hash value of one of the sequences appeared in the other. The HyperLogLog sketch^34^ is used to estimate the cardinality of the resulting hash sets. More precisely, the hash values are sorted into buckets via a predetermined prefix and the sketch value is given as the maximum leading zero count. We chose to set the length of the prefix to be 9, giving us a total of 512 buckets, or equivalently, features representing each genomic sequence.

Although Dashing can provide some form of privacy as it transforms raw genomic sequences into an abstract hash value, the process is not irreversible and thus not privacy-preserving.

### Differentially private federated model training

Due to a large amount of genomic data, we must select a sufficiently small subset of features that contains the most genomic information, to make the model training process tractable. Differential privacy is deployed during the training of local models before these models are combined into a global model.

#### Federated feature selection

The $\chi ^2$-test is a popular correlation test used to test the correlation between a feature and the response. A larger $\chi ^2$ value indicates that the feature and the response suggest a higher correlation and should be selected for the training of the model. In a traditional machine learning setting, feature selection is performed with the expectation that all the required data reside in the same machine. However, in this work, the data is split across several parties and cannot be directly shared, or pooled amongst the parties for feature selection. Thus a federated version of feature selection is necessary. We implemented a federated version of the $\chi ^2$-test proposed by Wang et al. in^5^. They proposed that in the federated setting, the $\chi ^2$-test can be approximated by its 2nd frequency moments. Based on our empirical testing, we find that a selection of 15 features is optimal.

#### Differentially private federated learning

We represented the sketch from Dashing as a one-hot vector and trained a logistic regression model using a differentially private SGD provided by Opacus. Opacus^35^ is a library that supports DP with PyTorch^36^. We used the cross entropy loss function with a learning rate of 0.01 and a default 60 training epochs. The value of $\varepsilon$ was varied and $\delta$ was set to $\tfrac{1}{D}$, where *D* is the total number of samples used to train the model.

### Homomorphic inference

The evaluation of the logistic regression model can be viewed as applying the sigmoid function on the inner product between the model weights and the features of the evaluating sample. As the sigmoid function serves to map the inner product output to probabilistic outcomes, we omit to evaluate the sigmoid function in the encrypted domain and instead determine the predicted class by choosing the largest value. We leverage the ability to store and operate on multiple data within a HE ciphertext to evaluate multiple samples simultaneously. The packing method we use in this implementation is based on^37,38^. The main idea would be to pack one feature from each sample into a single ciphertext. One plaintext–ciphertext multiplication is then performed and the resulting ciphertext is summed together to obtain the evaluation of the model on the new samples.

**Figure 4:**
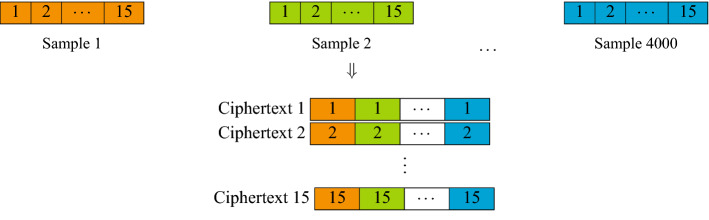
Encrypting samples. The same feature from all samples is encrypted into a single ciphertext. Since there are 15 features, a total of 15 ciphertext is used.

**Figure 5:**
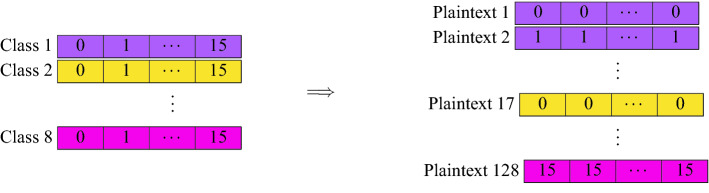
Encoding the model. Each feature is encoded multiple times in a single Plaintext object. A total of $8 \times 16=128$ Plaintext objects (15 features and 1 bias) is used.

**Figure 6:**
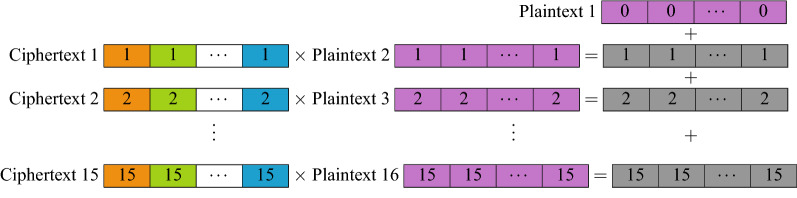
Homomorphic inference for a class. To evaluate if a sample belongs to a class, 15 plaintext–ciphertext multiplications are performed, followed by 15 summations of the resultant ciphertext and the bias Plaintext object. This allows all 4000 samples to be evaluated for a class simultaneously.

More precisely, the homomorphic inference first begins by Dashing the new samples, converting each sample into 64-bit hashes and the 15 chosen features are selected. The encryption process is shown in Fig. 4. The model, on the other hand, is encoded as depicted in Fig. 5. The homomorphic classification based on such an encoding method require Plaintext–Ciphertext multiplications and Ciphertext additions, described in Fig. 6.

## Supplementary Information

Supplementary Information.

## Data Availability

The data for the following strains-B.1.1.7 (Alpha), B.1.429 (Epsilon), P.1 (Gamma) and B.1.526 (Iota) are available from the organizers of the iDASH’2021 competition at http://www.humangenomeprivacy.org/2021/contact.html, which were used under license for the current study, and so are not publicly available. Data are however available from the corresponding author, Jun Jie Sim, upon reasonable request and with permission of the organizers of the iDASH’2021 competition. The data for the remaining strains - B.1.617.2 (Delta), C.37 (Lambda), B.1.621 (Mu) and B.1.1.529 (Omicron) are available in the GISAID repository, with Episet ID: $\mathtt {EPI\_SET\_220924cw}$ at https://doi.org/10.55876/gis8.220924cw.
