# Supplementary material for: CoVnita, an end-to-end privacy-preserving framework for SARS-CoV-2 classification
Source: Sci Rep. 2023 May 8;13:7461. doi: 10.1038/s41598-023-34535-8 (PMC10166033; doi:10.1038/s41598-023-34535-8)
Supplement: Supplementary file 1 — Supplementary Information. [file 41598_2023_34535_MOESM1_ESM.pdf]

Supplementary information

Data Split for Experiments

Party	Delta $B.1.617.2$	Lambda $C.37$	Mu $B.1.621$	Omicron $B.1.1.529$	Epsilon $B.1.429$	Alpha $B.1.1.7$	Gamma $P.1$	Iota $B.1.526$
1	2000							
2		2000						
3			2000					
4				2000				
5					2000			
6						2000		
7							2000	
8								2000

Table S1. Sample split where each party holds 1 **variant**. A total of 100 possible configurations were generated randomly.

Party	Delta $B.1.617.2$	Lambda $C.37$	Mu $B.1.621$	Omicron $B.1.1.529$	Epsilon $B.1.429$	Alpha $B.1.1.7$	Gamma $P.1$	Iota $B.1.526$
1	1000	1000						
2	1000	1000						
3			1000	1000				
4			1000	1000				
5					1000	1000		
6					1000	1000		
7							1000	1000
8							1000	1000

Table S2. Sample split where each party holds 2 **variants**. A total of 100 possible configurations were generated randomly.

Party	Delta $B.1.617.2$	Lambda $C.37$	Mu $B.1.621$	Omicron $B.1.1.529$	Epsilon $B.1.429$	Alpha $B.1.1.7$	Gamma $P.1$	Iota $B.1.526$
1	500	500	500	500				
2	500	500	500	500				
3	500	500	500	500				
4	500	500	500	500				
5					500	500	500	500
6					500	500	500	500
7					500	500	500	500
8					500	500	500	500

Table S3. Sample split where each party holds 4 **variants**. A total of 100 possible configurations were generated randomly.

Party	# Delta $B.1.617.2$	# Lambda $C.37$	# Mu $B.1.621$	# Omicron $B.1.1.529$	# Epsilon $B.1.429$	# Alpha $B.1.1.7$	# Gamma $P.1$	# Iota $B.1.526$
1	250	250	250	250	250	250	250	250
2	250	250	250	250	250	250	250	250
3	250	250	250	250	250	250	250	250
4	250	250	250	250	250	250	250	250
5	250	250	250	250	250	250	250	250
6	250	250	250	250	250	250	250	250
7	250	250	250	250	250	250	250	250
8	250	250	250	250	250	250	250	250

Table S4. Split where each party holds 8 **variants**. There is only one possible combination for such a split.

Continent	Delta $B.1.617.2$	Lambda $C.37$	Mu $B.1.621$	Omicron $B.1.1.529$	Total Samples
Africa	11	2	0	0	13
Asia	399	3	1	170	573
Europe	1175	54	32	1196	2457
North America	398	266	392	400	1456
Oceania	13	0	0	202	215
South America	4	1675	1575	32	3286

Table S5. Split according to geographical locations. The number of samples for each variant that was assigned to each continent is proportionate to the percentage of samples from that continent for the same variant.

Party	# Delta $B.1.617.2$	# Lambda $C.37$	# Mu $B.1.621$	# Omicron $B.1.1.529$	# Epsilon $B.1.429$	# Alpha $B.1.1.7$	# Gamma $P.1$	# Iota $B.1.526$	Total Samples
1	330	88	343	25	202	452	217	213	1870
2	195	125	376	36	341	131	260	244	1708
3	359	12	399	0	397	487	132	211	1997
4	406	613	107	109	316	456	251	152	2410
5	127	170	451	715	6	75	355	60	1959
6	318	487	54	293	400	195	298	244	2289
7	45	51	51	664	180	119	165	358	1633
8	220	454	219	158	158	85	322	518	2134

Table S6. Sample random split. Samples were randomly allocated amongst 8 parties using the uniform distribution. The allocation process is as follows: 8 random numbers r_i were first generated and the sum was calculated. The number of samples allocated to each party is then calculated with $\left\lfloor \frac{r_i}{\sum r_i} \times 2000 \right\rfloor$. A total of 100 possible configurations were generated randomly, of which 8 configurations had only seven out of eight variants.

Additional Model Performance Metrics

Setting	Data Distribution	# Variants per Party	Average Accuracy	Standard Deviation	2.5-th Percentile	97.5-th Percentile
Centralized	–	–	0.986	–	–	–
Federated	Balanced	1	0.873	0.149	0.508	0.978
		2	0.946	0.062	0.773	0.985
		4	0.980	0.008	0.959	0.987
		8	0.984	–	–	–
	Imbalanced	$2-4^{\star}$	0.994	–	–	–
		$7-8^{\#}$	0.975	0.016	0.946	0.989

Table S7. Model Accuracy for Centralized and Federated Settings. $(^{\star})$ refers to a split configuration based on geographical locations and $(^{\#})$ denotes a random split of samples across 8 parties.

Setting	Data Distribution	# Variants per Party	Average AUROC	Standard Deviation	2.5-th Percentile	97.5-th Percentile
Centralized	–	–	0.992	–	–	–
Federated	Balanced	1	0.978	0.033	0.894	0.999
		2	0.995	0.008	0.974	0.999
		4	0.999	0.001	0.997	0.999
		8	0.998	–	–	–
	Imbalanced	2 – 4*	0.999	–	–	–
		7 – 8#	0.998	0.001	0.994	0.999

Table S8. Model AUROC for Centralized and Federated Settings. (*) refers to a split configuration based on geographical locations and (#) denotes a random split of samples across 8 parties.

Setting	Data Distribution	# Variants per Party	Average APR	Standard Deviation	2.5-th Percentile	97.5-th Percentile
Centralized	–	–	0.974	–	–	–
Federated	Balanced	1	0.942	0.072	0.757	0.994
		2	0.988	0.010	0.958	0.997
		4	0.995	0.003	0.989	0.997
		8	0.992	–	–	–
	Imbalanced	2 – 4*	0.999	–	–	–
		7 – 8#	0.992	0.008	0.972	0.998

Table S9. Model APR for Centralized and Federated Settings. (*) refers to a split configuration based on geographical locations and (#) denotes a random split of samples across 8 parties.
